# Biomaterials and Regenerative Medicine in Pain Management

**DOI:** 10.1007/s11916-022-01055-5

**Published:** 2022-06-21

**Authors:** Xingjian Gu, Michelle A. Carroll Turpin, Mario I. Romero-Ortega

**Affiliations:** 1 Department of Biomedical Engineering, University of Houston Cullen College of Engineering, Houston, TX USA; 2 Department of Biomedical Sciences, University of Houston Tilman J. Fertitta Family College of Medicine, Houston, TX USA

**Keywords:** Chronic pain, Biopolymers, Growth factors

## Abstract

**Purpose of Review:**

Pain presents a unique challenge due to the complexity of the biological pathways involved in the pain perception, the growing concern regarding the use of opioid analgesics, and the limited availability of optimal treatment options. The use of biomaterials and regenerative medicine in pain management is being actively explored and showing exciting progress in improving the efficacy of conventional pharmacotherapy and as novel non-pharmacological therapy for chronic pain caused by degenerative diseases. In this paper we review current clinical applications, and promising research in the use of biomaterials and regenerative medicine in pain management.

**Recent Findings:**

Regenerative therapies have been developed to repair damaged tissues in back, joint, and shoulder that lead to chronic and inflammatory pain. Novel regenerative biomaterials have been designed to incorporate biochemical and physical pro-regenerative cues that augment the efficacy of regenerative therapies. New biomaterials improve target localization with improved tunability for controlled drug delivery, and injectable scaffolds enhance the efficacy of regenerative therapies through improving cellular migration. Advanced biomaterial carrier systems have been developed for sustained and targeted delivery of analgesic agents to specific tissues and organs, showing improved treatment efficacy, extended duration of action, and reduced dosage. Targeting endosomal receptors by nanoparticles has shown promising anti-nociception effects. Biomaterial scavengers are designed to remove proinflammatory reactive oxygen species that trigger nociceptors and cause pain hypersensitivity, providing a proactive approach for pain management.

**Summary:**

Pharmacotherapy remains the method of choice for pain management; however, conventional analgesic agents are associated with adverse effects. The relatively short duration of action when applied as free drug limited their efficacy in postoperative and chronic pain treatment. The application of biomaterials in pain management is a promising strategy to improve the efficacy of current pharmacotherapy through sustained and targeted delivery of analgesic agents. Regenerative medicine strategies target the damaged tissue and provide non-pharmacological alternatives to manage chronic and inflammatory pain. In the future, the successful development of regenerative therapies that completely repair damaged tissues will provide a more optimal alternative for the treatment of chronic pain caused. Future studies will leverage on the increasing understanding of the molecular mechanisms governing pain perception and transmission, injury response and tissue regeneration, and the development of new biomaterials and tissue regenerative methods.

## Introduction

Pain is an increasingly prevalent health problem affecting 1.5 billion people globally, with the number of adults reporting painful health conditions rising from 32.9% in 1998 to 41.0% in 2014 in USA alone [1]. The severity of this condition deleteriously impacts the quality of life or work activities in approximately 7.4% of the population [2]. Pain presents a unique challenge to treatment owing to both the complexity of the nociceptive signal transmission and modulation, and the high variability of pain perception among individuals. Treatment goals include reduced noxious sensation and improved function, and the specific strategy depends both on the severity and the temporal nature of the pain, and the needs of the patient.

For many years, the World Health Organization (WHO) Pain Ladder, which was initially developed for the treatment of cancer pain, was a simple straightforward tool that has been used to guide both cancer and non-cancer pain management, recommending nonsteroidal anti-inflammatory drugs (NSAIDs) for mild cases (step 1: non-opioids), followed by opioids for moderate and severe conditions (steps 2 and 3) [3].

The use of opioids for pain management is not without its drawbacks. Opioids produce a number of adverse effects ranging from the unpleasant, such as nausea, vomiting, and constipation, to life-threatening respiratory depression. Moreover, opioid use runs the risk of physical and psychological dependence. Between 21 and 29% of patients taking opioids for chronic pain misuse their opioids and approximately 10% develop opioid use disorder [4]. Opioid over-prescribing for pain is considered an initial driving factor for the Opioid Epidemic. Although the rate at which opioids are prescribed peaked in 2012, over 142 million opioid prescriptions were dispensed in 2020 [5]. Despite the decline in prescription opioid use, US opioid overdose deaths continue to rise [6].

As the use of prescription opioids decreases, the necessity of identifying novel, non-opioid pain management options grows. For example, even with the use of local anesthetics and analgesics, it is estimated that 39% of patients who undergo surgery do not have adequate postoperative analgesia and experience mild to severe pain [7]. Moreover, when it comes to the treatment of chronic pain, the average patient can expect only about a 30% reduction in their pain score [8].

Recently, the WHO Pain Analgesic Ladder has been revised to help reduce the role of opioids so that the risk of misuse and dependency might be minimized. The new version allows for the inclusion of non-pharmacological treatment strategies and suggests a bidirectional approach for pain, starting at the bottom and scaling up to manage chronic pain, versus starting with the strongest agent (appropriate for the severity) then working down from there. Additionally, a new step 4 outlines invasive or minimally invasive treatments, including sustained analgesia delivery methods, neuromodulation, nerve block, and ablation therapies [3]. Innovation in biomaterials, biomolecular controlled release, and regenerative medicine are providing new clinical alternatives for pain treatment.

Biomaterial and regenerative medicine are rapidly growing research fields, their potential application to pain management is being actively explored, and significant progress has been made in the last decade. Novel regenerative therapies have been developed to repair degenerated tissues that lead to chronic and inflammatory pain in back, joint, and shoulder, with the potential to identify and eliminate the source of pain. Pain management biomaterials are developed to serve as drug carriers that target specific tissues, cell types, and organelles with sustained, localized, and stimuli-responsive release of pain medication, exhibiting improved efficacy and longer-term relief of pain symptoms. Biomaterial scavengers are designed to remove proinflammatory reactive oxygen species that trigger nociceptors and cause pain hypersensitivity, providing a proactive approach for pain management. Below, we explore and discuss the different scenarios that biomaterials and regenerative medicine can be applied to pain management and present recent progress in the use of biomaterials for chronic pain management.

## Regenerative Medicine in Pain Management

Regenerative medicine has the potential to manage or cure pain resulting from tissue injury or inflammation without the continuous use of analgesics. Regenerative therapies including biomaterials, engineered tissues, and medical devices have been developed to support, repair, or replace damaged or abnormal tissues, restore their healthy state, and relieve the associated pain. Currently, regenerative therapies can be used to treat back pain arising from degenerative intervertebral disks (IVD) [9], knee pain caused by osteoarthritis and meniscus degeneration, shoulder pain that results from damaged rotator cuff [10], jaw pain from damaged temporomandibular joint (TMJ) [11], tendinitis pain from a tendon injury, and neuropathic pain from irritated, damaged or inflamed nerves [12].

### Regenerative Biomaterials

Biomaterials used to promote tissue regeneration include bioactive ceramics; natural polymers such as chitosan, hyaluronic acid, and collagen; and synthetic polymers such as polycaprolactone (PCL) and poly(lactic-*co*-glycolic acid) (PLGA) [13]. These materials have shown great promise in bone and cartilage repair [14, 15], and in nerve regeneration [16], and provide a microenvironment that augments the regenerative potential of both the transplanted and host cells [17, 18]. Scaffold pore architecture regulates chondrogenesis and endochondral ossification of bone marrow–derived mesenchymal stem cells (BMSCs) and promotes vascularization [19], and alignment of extracellular molecule hydrogels promotes myotube formation of myoblasts [20]. Polycaprolactone (PCL)–based nano-topographic patches with aligned nanoscale matrix (ridges and grooves of ~ 800 nm) with nanosized pores (~ 100 nm), promote the proliferation and osteogenic mineralization in vivo [21].

The use of regenerative biomaterials for pain management has been extensively studied in IVD, which is the leading cause of low back pain, where PCL microfiber scaffolds, collagen peptide (Pro-Hyp-Gly)-presenting hydrogels, and adipose mesenchymal stem cell–derived tissue-engineered constructs have been tested [9, 22, 23]. For joint pain caused by articular cartilage and meniscus degeneration, hyaluronan scaffolds grafted with biomimetic brush-like nanofibrous polymers improved osteoarthritis within 8 weeks in a rat model by forming a lubrication layer on the cartilage surface [24]. Additionally, ECM scaffolds conjugated with aptamer HM69, viscoelastic PEGylated poly(glycerol sebacate) scaffolds combined with the osteoinductive mesoporous bioactive glass (MBG), and BMSC-laden biomimetic multiphasic scaffolds have shown to be effective in tissue regeneration [25–27]. Similarly, functionally graded scaffolds with anisotropy properties mimicking its hierarchical microstructure have shown superior repair outcomes in rotator cuff injury which often causes shoulder pain [28–30]. These novel biomaterials have the potential to enhance the regeneration and regulate the inflammation status of the diseased tissue, provide substantial alleviation or elimination of the pain symptoms associated with these diseases, and thus serve as a good alternative or supplement to current pharmaceutical therapies for pain management.

### Controlled Release of Regenerative Therapeutics

Bioactive agents such as growth factors and platelet-rich plasma (PRP) have shown great promise in regenerative medicine due to their anti-inflammatory effects and the ability to activate the intrinsic regenerative pathways. However, direct injection without a delivery system results in significant loss of the therapeutic agents due to leakage, diffusion, denature, and circulatory clearance. Drug delivery systems can be used to carry therapeutic agents and release them in a more controllable manner. By tuning the material composition and physical structure of the delivery system, desired release patterns can be achieved.

Controlled release of growth factors [31–33], mesenchymal stem cells [34–36], nucleotides [37], and exosomes [38] have shown potential in promoting tissue regeneration due to the unparalleled pro-regenerative bioactivity of these substances. Matrilin-3, a non-collagenous protein, has been found to improve regeneration of articular cartilage by maintaining chondrogenesis and preventing the hypertrophic transition of BMSCs in an ECM mimicking nanofibrous scaffold [39, 40]. Advanced composite systems are composed of hyperbranched polymer, PLGA nanoparticle, and spongy PLA microsphere developed to simultaneously deliver anti-miR-199a and mesenchymal stem cells for IVD regeneration [37]. Branched poly(ester urea) (PEU) nanofibers have also been used to adsorb and retain PRP at the implant site for enhanced rotator-cuff repair [41]. For local accumulation and protection of the growth factors against degradation, heparin has been incorporated in the delivery system to deliver stromal cell–derived factor-1α and growth and differentiation factor-5 for IVD regeneration [32, 42]. Other advanced drug release methods include a ligand-modified delivery system for permeation across physiologic barriers [43], microtechnology or microelectromechanical systems (MEMS)–based delivery technology for spatial-, temporal-, and dosage-controlled release [44], magnetic or electric field–sensitive delivery systems for biosensing, and bioimaging [45].

### Injectable Scaffolds

**Fig. 1:**
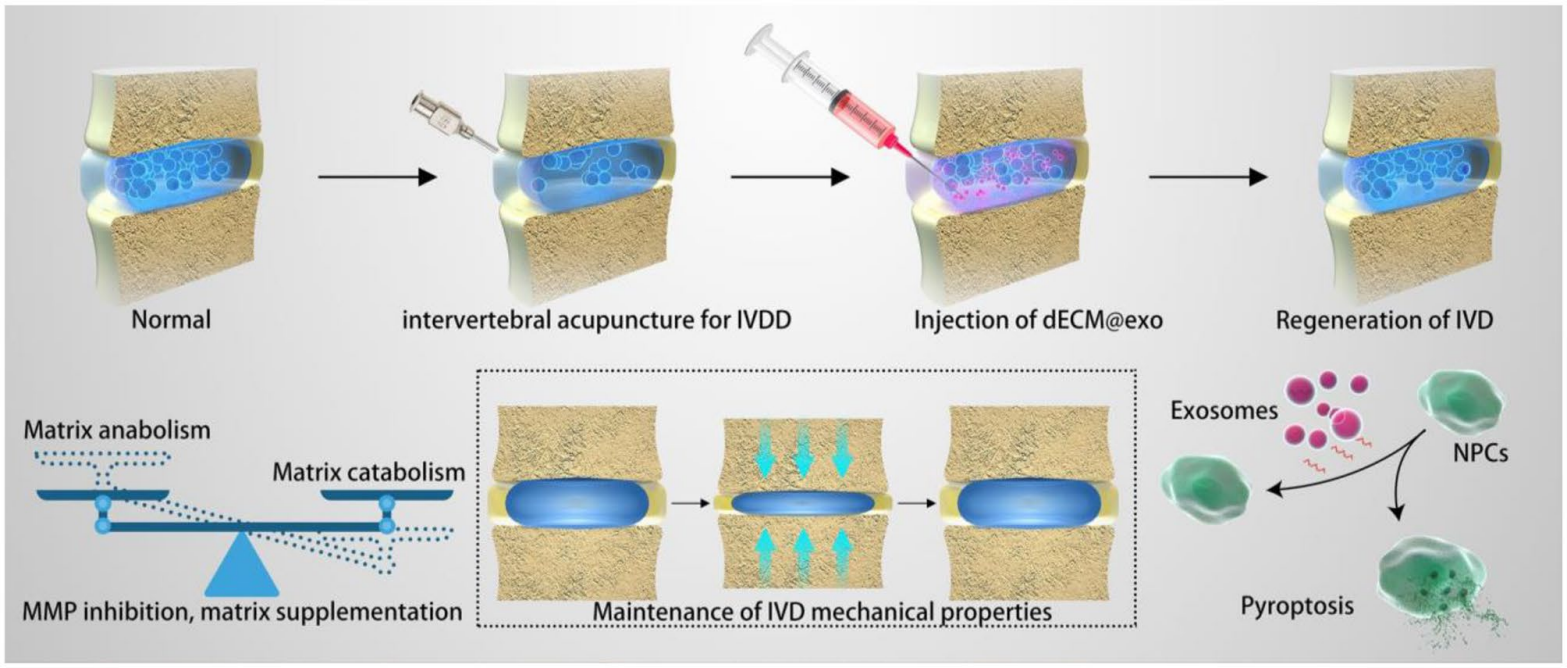
Thermosensitive acellular extracellular matrix (ECM) hydrogel coupled with adipose mesenchymal stem cell (ADSCs) exosomes for IVD regeneration [38]. Sustained release of ADSC-derived exosomes regulates matrix synthesis and degradation by regulating matrix metalloproteinases (MMPs) and inhibits pyroptosis by mitigating the inflammatory response. Reproduced with permission

Injectable systems have been proposed for both tissue repair and localized drugs using gel matrices and nano- or microparticles. Hydrogel scaffolds can be directly injected in their liquid state using a syringe and then undergo in situ gelation to form a solid scaffold. Recently, injectable scaffolds have been fabricated from various biomaterials for IVD and osteochondral regeneration [36, 46–50]. The injectability of tissue engineering scaffolds can reduce the tissue damage caused by the transplant surgery, simplify the procedure, and lower the cost. Also, injectable scaffolds can serve as the delivery platform for local administration and controlled release. Collagen shows excellent biocompatibility and bioactivity, and decellularized extracellular matrix (ECM) obtained from fresh nucleus pulposus (NP) tissue has been used as an injectable scaffold to deliver adipose-derived stem cells exosomes for IVD regeneration (Fig. 1) [38]. A self-assembling β-sheet forming octa-peptide with alternative hydrophilic and hydrophobic residues has been proposed as an injectable scaffold for IVD regeneration with tunable mechanical properties to match with NP tissue and good injectability to deliver NP cells and growth factors [36, 48]. Other novel strategies for self-assembling systems have also been reported, such as host–guest interactions [51], metal–ligand coordination [52], and dynamic covalent reactions [53].

## Pain Management Biomaterials

### Sustained Release of Pain Medication

Biomaterials have been used to fabricate novel drug carriers to encapsulate pain drugs for local and controlled delivery. As traditional formulations for post-operative and chronic pain have a short duration of effect, sustained-release drug carriers have been developed using biodegradable polymers, lipids, and mesoporous silica, in the form of microparticles and nanoparticles, liposomes, micelles, and dendrimers [54, 55]. Analgesics, including opioids, local anesthetics, NSAIDs, neuropeptides, cannabinoid, and neurotoxins, have been encapsulated in biocompatible materials such as chitosan and PLGA for sustained release [56]. Site 1 sodium channel blocker tetrodotoxin (TTX) is a potent nerve blocker, and sustained and tunable TTX release can be achieved by conjugating it to the biodegradable polymer poly(triol dicarboxylic acid)-co-poly(ethylene glycol) [57], from several hours to 3 days, with minimal systemic or local toxicity. PEG-PLGA microparticles have been used for ketamine sustained release, 21 days in vitro and 5 days after intravenous injection (1 mg/kg) in mice [58]. In 2020, the FDA approved the use of Posimir™ (Durect Inc), bupivacaine-impregnated poly(lactide-caprolactone) microparticles designed as an injectable, that allows 72 h of extended co-release of bupivacaine and a low dose of NSAID meloxicam, for arthroscopic subacromial decompression [55].

Recently, the FDA approved the use of Zynrelef™ (Heron Therapeutics) that provides 72-h release of bupivacaine-meloxicam using a polydioxanone polymer carrier [59] and of XaraColl® (Innocoll Pharmaceuticals), which is a non-injectable collagen implant containing bupivacaine, for 24 h of pain relief after hernia surgery [60, 61]. These novel biomaterial-based pain management formulations significantly reduced post-operative pain and opioid use in patients undergoing bunionectomy, herniorrhaphy, or total knee arthroplasty [62].

### Targeted Delivery Strategy for Pain Management

Development of novel carriers that deliver drugs to a specific body site can increase the drug concentration at the site of interest and limit the systemic exposure to the drug, thereby enhancing treatment efficacy and reducing side effects. This is especially beneficial for patients with localized pain and chronic pain. Site-specific delivery can be achieved by modification of biomaterials with targeting molecules such as peptides and antibodies. For example, liposomes anchored with acylated integrin-targeting peptides (palmitoyl–Gly–Arg–Gly–Asp–Ser) were developed for nasal delivery of fentanyl, which showed stability under aerosolization, enhanced central nervous system analgesic effects, and reduced plasma drug exposure [63]. More recently, in an animal study, conjugation of liposomes with antibodies that recognizes an extracellular domain of the oxytocin receptor increased the localization of the liposomes at the uterus by sevenfold [64]. This immunoliposome strategy was used to effectively deliver indomethacin for the prevention of inflammation-induced preterm labor in pregnant mice, with reduced dose and reduced toxicity to both mother and fetus.

Brain-targeting carriers have been developed to enhance drug penetration across blood–brain barrier which has the potential to improve the analgesic response while maintaining, or reducing, dose and minimizing adverse side effects. The analgesic potency of the morphine metabolite morphine-6-glucuronide (M6G) is 50-fold higher than morphine when administered via intracerebral injection, but the significantly lower brain penetration of M6G following more conventional delivery methods limits its application in pain management [65]. Eiselt and colleagues conjugated M6G with a brain-targeting peptide angiopep-2 peptide (An2), which crosses the blood–brain barrier by low-density lipoprotein receptor-related protein 1 (LRP1)-receptor mediated transcytosis and demonstrated significantly improved brain penetration and analgesic potency of M6G. The An2-M6G conjugate also showed a favorable side-effect profile that includes reduced likelihood of developing constipation.

Targeting endosomal receptors that mediate nociception using nanomaterials has been proposed as a promising pain management strategy. In chronic pain, the substance P (SP) neurokinin 1 receptor (NK_1_R) redistributes from the plasma membrane to acidified endosomes, where it signals to maintain pain [66]. Ramírez-García and colleagues developed novel pH-responsive polymeric nanoparticles to precisely deliver FDA-approved NK_1_R antagonist aprepitant and inhibit endosomal NK_1_R signaling. Intrathecal injection of these nanoparticles induced a more complete and persistent relief from nociceptive, inflammatory, and neuropathic nociception in preclinical models than that of opioids. In another study, the δ-opioid receptor (DOPr) agonist [D-Ala(2)-D-Leu(5)]enkephalin (DADLE) was encapsulated into mesoporous silica nanoparticle core (lipoMSN), to selectively target DOPr-expressing neurons and activate their endosomal DOPr for relief from inflammatory pain [67]. One intrathecal injection of the lipoMSN provided an analgesic effect lasting for 6 h in a mouse model of inflammatory nociception. These novel nanomaterials that selectively direct drugs to subcellular compartments open the opportunity for developing much-needed non-opioid therapies for pain.

### ROS Scavenging Biomaterials

Reactive oxygen species (ROS) are byproducts of cellular functions such as oxidative phosphorylation. In pathological conditions, excess ROS accumulates and causes inflammation, cell and tissue damage, and pain [68]. It has been shown that pro-inflammatory microglial activation with aberrant ROS generation in the spinal cord plays a critical role in the development of neuropathic pain [69]. To manage neuropathic pain by reducing ROS levels in microglia, Choi and colleagues developed a novel microglia-targeting ROS scavenging nanomaterial by conjugating microglia-specific antibody CD11b to ceria-zirconia nanoparticles [70]. The targeted delivery facilitated the elimination of both pro-inflammatory cytokines and ROS in microglia and ameliorated mechanical allodynia in a spinal nerve transection-induced neuropathic pain mouse model. Other novel ROS scavenging materials have been proposed for various applications, including ceria nanocrystals decorated mesoporous silica nanoparticles [71], movable hemin-loaded mesoporous silica nanoparticles [72], poly(NIPAAm-co-VP-co-MAPLA-co-MATEMPO) hydrogel [73], and enzyme-mimicking ultrasmall Cu_5.4_O nanoparticles [74]. Table 1 lists current research a clinical application of biomaterials for pain management.

**Table 1:** Pain management biomaterials

Application	Biomaterials	Analgesics	Major results	Ref
Sustained delivery	Poly(triol dicarboxylic acid)-co-poly(ethylene glycol)	Tetrodotoxin	1.0–80.0 µg tunable range of nerve block duration, from several hours to 3 days, with minimal systemic or local toxicity	[57]
Sustained delivery	PEG-PLGA microparticles	Ketamine	Sustained release for 21 days in vitro and 5 days after intravenous injection	[58]
Targeted delivery: uterus	Liposomes conjugated with anti-oxytocin receptor antibody	Indomethacin	Increased localization to the uterus by sevenfold; for the prevention of inflammation-induced preterm labor pain	[64]
Targeted delivery: brain	Angiopep-2 peptide	Morphine-6-glucuronide	Greater and more sustained analgesic activity than equivalent doses M6G	[65]
Targeted delivery: endosome	Composite polymeric nanoparticles, encapsulating aprepitant	N/A	Inhibited substance P-induced activation of spinal neurons; complete and persistent relief from nociceptive, inflammatory, and neuropathic nociception	[66]
	Mesoporous silica nanoparticle core with DADLE-conjugated liposome shell	N/A	Achieve long-lasting activation of DOPr in endosomes; provided sustained inhibition of nociceptor excitability and relief from inflammatory pain	[67]
ROS scavenging	Ceria-zirconia nanoparticles conjugated with anti-CD11b antibody	N/A	Facilitated elimination of both pro-inflammatory cytokines and ROS in microglia; greatly ameliorated mechanical allodynia in neuropathic pain	[70]

## Limitations and Future Direction

Currently, the application of regenerative medicine strategy in pain management mainly focuses on IVD degeneration, as it is the leading cause of low back pain. The association of other tissue injury and degenerative diseases with the development of pain symptoms received less attention. In addition, most of the studies investigating regenerative therapies did not report the efficacy of pain relief compared with other, more traditional, pain management strategies, which is needed to fully evaluate the benefit of these therapies. In future studies, establishing experimental standards to evaluate pain relief and conducting controlled experiments to include pharmacotherapy-only groups are needed to develop regenerative medicine alternatives for pain management. New understanding of the pathophysiology of other types of chronic and inflammatory pain will lead to the development of novel therapies to treat those pain generators. Given the intrinsic variability and complex regulatory network in tissue injury and regeneration, identifying the appropriate combination of bioactive molecules, optimal concentrations, and delivery timing represents a significant challenge. More detailed understanding of cell-environment, cell–cell, and intracellular signaling events during tissue injury response and regeneration process is a prerequisite to develop more effective regenerative therapies. This can be achieved by conducting research to find the key molecules to target, leveraging latest innovations in single-cell technologies, multi-omics, and computational analysis methods such as machine learning. In addition, the biocompatibility of regenerative biomaterials used in tissue repair needs to be refined to reduce the foreign body response and adapted for personalized use according to patient-specific factors such as obesity and diabetes [75].

Drug delivery systems encapsulating analgesics significantly enhanced their efficacy and duration of effect. However, these biocompatible and biodegradable polymeric materials are not compatible with current imaging methods to determine biomaterial-drug biodistribution and local drug concentration [77]. Biomaterials designed to incorporate imaging capability will provide prognostic value for these drug delivery platforms and increase the precision of pain medication delivery [76]. Apart from this, considerable effort will be required to advance these compounds to the clinic, including include toxicology, pharmacokinetic, and pharmacodynamic studies in disease-relevant preclinical models. The therapeutic efficacy of these formulations could be improved by encapsulating antagonists of different targets that co-mediate pain transmission and their signaling pathways [77].
